# Interest in a short psychological intervention in patients with psoriasis: a cross-sectional observational study at a German clinic

**DOI:** 10.3389/fmed.2023.1074632

**Published:** 2023-06-15

**Authors:** Laura R. Stadtmüller, Markus A. Eckardt, Christoph Zick, Joerg Kupfer, Christina Schut

**Affiliations:** ^1^Institute of Medical Psychology, University of Giessen, Giessen, Germany; ^2^Department of Dermatology, Rehabilitation Clinic Borkum Riff, Borkum, Germany

**Keywords:** psoriasis, anxiety, depression, illness perception, psychological intervention

## Abstract

**Introduction:**

Utilization of health services is not only associated with the kind of illness one has, but also with patient characteristics like age, sex or psychological variables. Psoriasis (PS) is a chronic inflammatory skin condition, in which psychological interventions were shown to be beneficial regarding not only psychological variables, but also regarding the skin status. The present study investigated with regard to which patient characteristics PS-patients with interest in participation in a short psychological intervention differ from PS-patients without interest.

**Methods:**

This cross-sectional questionnaire study was conducted at a German rehabilitation clinic. At the beginning of their stay at the clinic, 127 PS-patients filled in questionnaires to assess the severity of their PS, stress, illness perceptions, mindfulness, anxiety, and depression. Interest in taking part in a short psychological intervention was assessed using a dichotomous item. The statistical analysis comprised group comparisons using *t*-tests of patients with and without interest to take part in a short psychological intervention.

**Results:**

Sixty-four of the participants were male (50.4%). Participants were 50.7 ± 10 years on average (range: 25–65). 50.4% of them had a mild, 37.0% a moderate, and 12.6% a severe PS. Results indicated that patients with interest in a short psychological intervention were younger, reported to have more skin symptoms due to their PS (higher skin-related illness identity), were more anxious and depressed, but less stressed and less mindful than patients without interest.

**Conclusion:**

This study shows that in PS-patients with certain characteristics, it might help to raise awareness on the relationship between psychological factors and symptoms of the skin disease in order to motivate this group of patients to take part in psychological interventions to improve their skin condition. Further studies are needed to investigate whether patients who show interest in a psychological intervention also actually take part in the intervention and profit from it.

**Clinical Trial Registration:** DRKS00017426.

## Introduction

The utilization of health services is related not only to disease-related factors [e.g., self-rated health, multimorbidity (1, 2)], but also to sociodemographic variables such as age, sex and socioeconomic status (2, 3). Having a psychological disease like an anxiety disorder, depression, panic disorder, somatoform disorder, or affective disorder as comorbidity is associated with higher use of health-related services (4, 5).

Psychological interventions, which can be a useful add-on in the treatment of different conditions, are more often used by women, persons without a partner or job, and patients with chronic diseases compared to persons with the opposite characteristics (3, 6, 7). Also, complementary and alternative medicine are used more often by women (8).

Psoriasis (PS) is a chronic, inflammatory disease with a 1-year-prevalence of about 2.5% in the German population (9). In other studies prevelance rates between 0.9 % and 8.5% were found (10). It is a multifactorial condition in which psychological factors such as stress (11), anxiety (12), and depression (13, 14) play a role.

In PS patients, psychological interventions such as meditation or cognitive behavior therapy were shown to have effects on the severity of PS symptoms (15). Also, mindfulness-based cognitive interventions often, but not always led to a significant improvement in the severity of the skin condition and a better quality of life (16–19).

Thus, psychological interventions seem to be beneficial in patients with PS and especially in those who experience psychological stress in daily life (20). However, in this patient group demographic, skin-related and psychological variables associated with interest in such a psychological intervention have not been identified, although interventions should be adapted to the needs of the patients (21). The aim of the study therefore was to examine if patients with and without interest in a short psychological intervention differ regarding demographic, psychological, and skin-related factors.

## Methods

### Study design and setting

This observational study took place at the rehabilitation clinic Borkum Riff, a clinic for patients with skin conditions and pneumological diseases. In Germany, a stay at a rehabilitation aims to reduce the severity of a disability or prevent its aggravation in order to reduce days of absence from work. Usually, patients stay there for 4 to 6 weeks. Data collection started in August 2019 and ended in September 2020. There were three data collection periods: August–September 2019; March 2020; and July–August 2020. Patients were recruited during their first week at the clinic by two of the authors (LS; ME) and a psychology student (see Acknowledgements). The study protocol was published before the recruitment of the participants was completed [for further details see Stadtmüller et al., (22)].

### Participants

Patients were included consecutively. They were eligible to take part, if they fulfilled the following inclusion criteria: age between 18 and 65 years, clinical diagnosis of PS according to the International Classification of Diseases ICD-10 (23) for at least 6 months as well as the occurrence of symptoms during the last 6 months and sufficient knowledge of the German language in order to be able to fill in the questionnaires. They were excluded in case they were cognitively impaired. We originally planned to exclude patients with concomitant other skin conditions (especially itchy ones) than PS, but during the process of the study decided to include them. However, we conducted separate analyses for the group of patients with and without another skin condition.

### Variables

#### Quasi-dependent variables

Demographic variables (age and sex), severity of PS, perceived stress, illness perception, anxiety, depression, and mindfulness were assessed as variables possibly distinguishing between patients with and without interest in a short psychological intervention.

The severity of PS was measured by the Self-Administered Psoriasis Area and Severity Index (SAPASI), which is a validated instrument that records the severity of PS by assessing the intensity of redness, thickness, and scaliness of the skin as well as the extent of affected areas (24).

The perceived stress level was measured by the Perceived Stress Scale [PSS; (25, 26)] which comprises 10 items (e.g., “How often have you been feeling nervous and stressed during the last week”) that need to be answered on a 5-point scale. In this study, we were interested in the stress level during the last week instead of during the last month and therefore modified the wording in the instruction accordingly [also see Stadtmüller et al. (22)].

Furthermore, patients’ illness perceptions were assessed with the German version of the Illness Perception Questionnaire (IPQ) capturing the five dimensions disease illness identity (e.g., “How frequently have you experienced pain as part of your illness”; divided into skin related and general illness identity), experienced causes of the disease (e.g., “A germ or virus caused my illness”), time-line of the disease (e.g., “My illness will last a short time”), consequences of the disease (e.g., “My illness is a serious condition”), and cure control (e.g., “My illness will improve in time”; 27).

Anxiety and depression were measured by means of the Patient Health Questionnaire (PHQ), which includes four items, two measuring the cardinal symptoms of anxiety disorders and two measuring the cardinal symptoms of depression (28).

After internal discussion within the working group, the Comprehensive Inventory of Mindfulness Experience (CHIME; 29) was additionally used to assess current levels mindfulness as a variable potentially differing between patients with and without interest in a short psychological intervention.

#### Quasi-independent variable

Interest in a short psychological intervention was assessed by the dichotomous item “Are you interested in participation in a short psychological intervention during your stay at the rehabilitation clinic?” The explanation that the intervention would be a mindfulness-based training at the clinic was orally added as the word psychological intervention led to resistance at the beginning of the study.

#### Further variables

Further sociodemographic variables such as education level, family status, and itch (average and maximal itch intensity during the last 24 h) were assessed in order to describe the sample. For more information, see Stadtmüller et al. (22).

### Statistical analysis

As mentioned before, we conducted two analyses, one including only patients without any other itchy skin disease than PS (*n* = 111) and another one including all patients (*n* = 127), also the ones who had another itchy skin condition besides PS (*n* = 16). Because the results of the two analyses did not differ significantly, we will only present the results of the analyses including all patients in this manuscript. The statistical analysis was done using SPSS version 28 (30). To investigate whether patients with and without interest in a short psychological intervention differed, *t*-tests for independent groups were conducted.

### Ethics

The study was conducted in concordance with the declaration of Helsinki. The local ethics committee of the Faculty of Medicine at the Justus-Liebig-University approved the study (date of IRB approval: March 21st, 2019; AZ 19/19). In addition, the Federation of German Pension Insurance Institutions (Deutsche Rentenversicherung Bund, DRV-Bund) approved the study before recruiting the first study participant. All eligible patients were informed about the purpose and procedure of the study. Subjects participated in the study on a voluntary basis and were free to withdraw from the study at any time. They received a monetary allowance of 15 € for participation. The data were collected pseudonymously and kept locked separately from the consent forms.

## Results

### Participants

**Figure 1:**
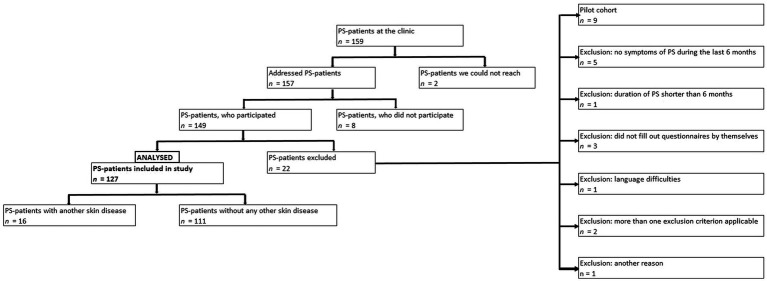
Overview of included and excluded patients and exclusion criteria.

One hundred and fifty-nine PS-patients were treated at the rehabilitation clinic the during data collection periods. One hundred and fifty-seven persons could be reached, 149 patients took part in the study. 127 patients could be included in the analyses, while 22 had to be excluded *post hoc* because of not fulfilling the inclusion criteria (see Figure 1).

### Sample characteristics

Sex was equally distributed in the sample: *n* = 64 of the participants were male (50.4%), *n* = 63 were female (49.6%). Participants were 50.7 ± 10 years on average (range: 25–65). 99.2% were German. More than half of the participants were married (53.5%) and living with their husband/ wife (55.1%). 60.6% had no possibility to go to college, 9.4% had this possibility, and 29.9% had a university diploma. The mean duration of PS was 20.8 ± 13.5 years (range: 0.5–54 years) with an average SAPASI of 7.3 ± 4.1 (range: 0.7–19.4) at the time of assessment. According to the SAPASI-classification (24), 64 (50.4%) had a mild PS, 47 (37.0%) a moderate and 16 (12.6%) a severe PS. Forty-seven participants did not have interest in a short psychological intervention, while 80 had interest. For further sample characteristics, see Table 1. A more detailed description of the sample will be given in the doctoral thesis by the first author of this article.

**Table 1:** Sample characteristics.

Variable	Subscale	x̄	SD	Range
Illness perception	IPQ_A_illness_identity_general	5.5	2.8	0–10
Illness perception	IPQ_D_illness_identity_skin	3.8	3.0	0–11
Illness perception	IPQ_cause_stress	3.7	1.1	1–5
Illness perception	IPQ_cause_nutrition	3.2	1.1	1–5
Illness perception	IPQ_cause_mental_state	3.1	1.3	1–5
Illness perception	IPQ_cause_timeline	4.1	0.8	1–5
Illness perception	IPQ_cause_consequenses	2.9	0.7	1–5
Illness perception	IPQ_cause_control/cure	3.2	0.6	1–5
Stress	PSS _total	2.3	0.7	0–4
Anxiety	PHQ_Anxiety	0.9	0.8	0–3
Depression	PHQ _Depression	0.9	0.7	0–3
Mindfulness	CHIME_total	3.9	0.5	2.8–5.1

SD, Standard deviation; x̄, mean; IPQ, Illness perception questionnaire; PSS, Perceived Stress Scale; PHQ, Patient Health Questionnaire; CHIME, Comprehensive Inventory of Mindfulness Experiences.

### Differences between patients with and without interest

**Figure 2:**
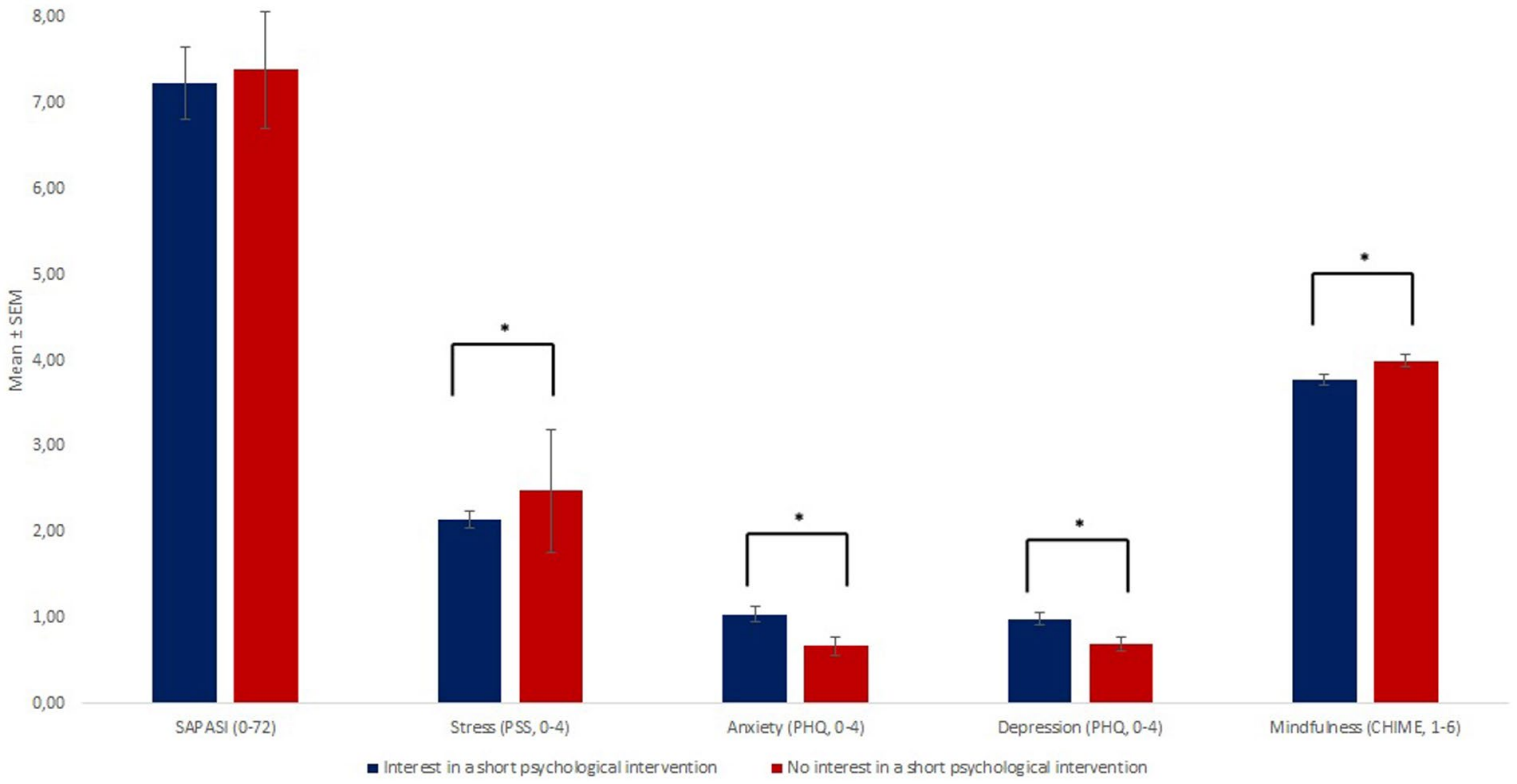
There were no group differences regarding the skin status, but regarding stress, anxiety, depression and mindfulness between patients with and without interest in a psychological intervention. * Illustrates a significant group difference (*p*<0.05).

**Figure 3:**
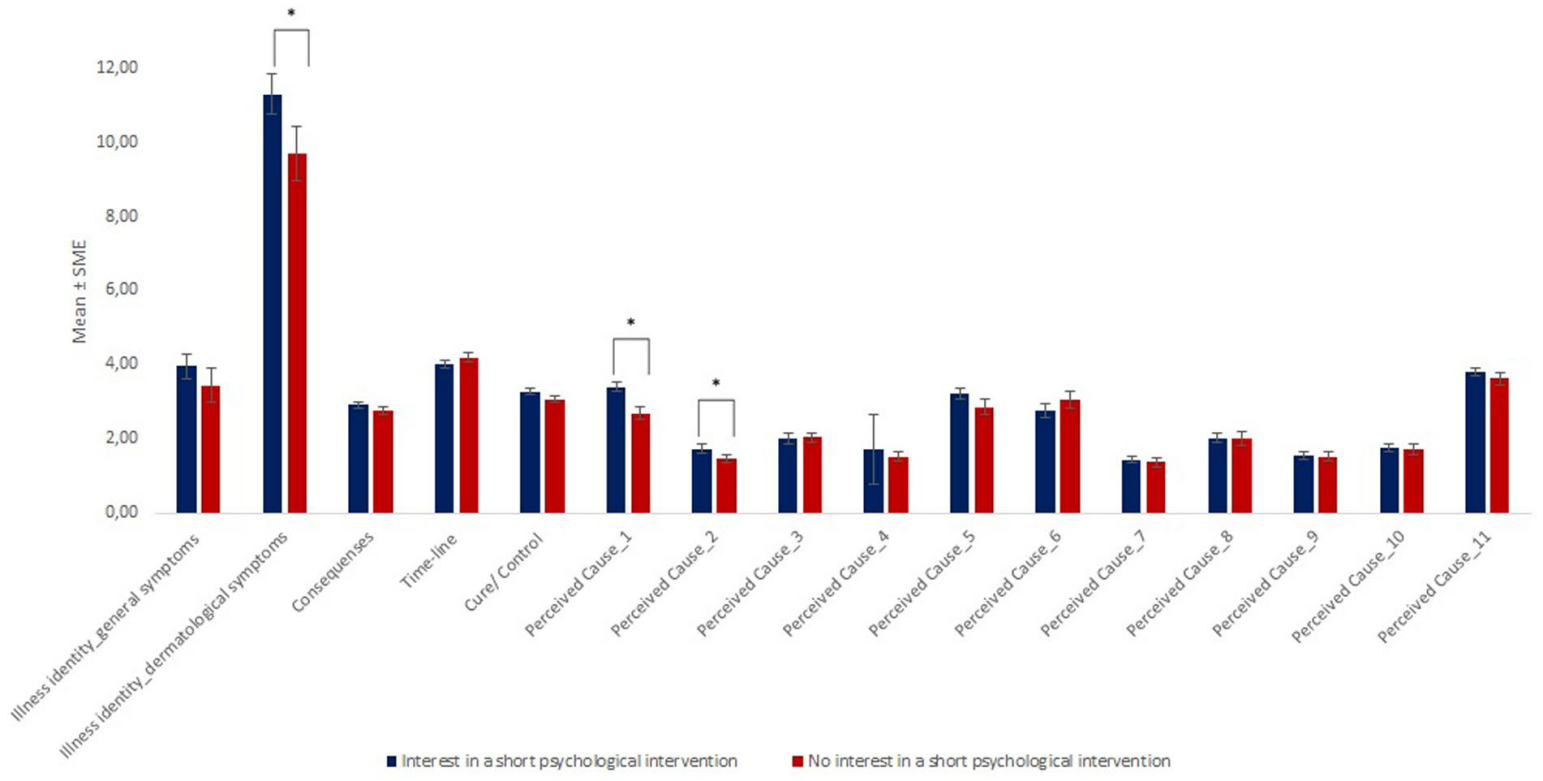
Group differences regarding illness perception. Explanations: Perceived Cause_1: Nutrition played a major role in causing my illness; Perceived Cause_2: A germ or virus caused my illness; Perceived Cause_3: It was just by chance that I became ill; Perceived Cause_4: Pollution of the environment caused my illness; Perceived Cause_5: My state of mind played a major part in causing my illness; Perceived Cause_6: My illness is hereditary—it runs in my family; Perceived Cause_7: Allergy causes my illness; Perceived Cause_8: Other people played a large role in causing my illness; Perceived Cause_9: My illness was caused by poor medical care in the past; Perceived Cause_10: My illness is largely due to my own behavior; Perceived_Cause_11: Stress was a major factor in causing my illness. * Illustrates a significant group difference (*p*<0.05).

*T*-Tests for independent groups showed significant differences between patients with and without interest in a short psychological intervention regarding age, illness identity, stress, anxiety, depression, and the belief that nutrition and virus/bacteria cause the disease as well as mindfulness [*p* < 0.05]: Patients with interest in a short psychological intervention were younger [*p* = 0.031], reported to have more skin symptoms due to their PS [IPQ-scale skin-related illness-identity; *p* = 0.041], reported to be more anxious [*p* = 0.005], more depressed [*p* = 0.01], less stressed [*p* = 0.006], and less mindful [*p* = 0.008] than patients who were not interested in the intervention. In addition, patients with interest in a short psychological intervention more often believed that nutrition and virus/bacteria are an important cause of the disease than patients without interest [nutrition: *p* < 0.001; virus/bacteria: *p* = 0.034]. For further details, see Figures 2, 3.

## Discussion

The aim of this study was to analyze how PS-patients with and without interest in participation in a short psychological intervention differ regarding demographic variables, the severity of PS, illness perception, anxiety, depression, stress and mindfulness. Knowledge regarding factors contributing to interest in participation in a short psychological intervention is necessary in order to offer patients individual information about the advantages and disadvantages of psychological interventions and their effects.

The study revealed that interested patients were younger, reported to have more skin symptoms due to their PS, be more anxious, more depressed, but less stressed and less mindful than patients without interest in a short psychological intervention. Interested persons also more often regarded nutrition and a virus/bacteria as cause of their disease than non-interested persons.

These findings are partly in congruence with other studies, in which more anxious and depressed patients also had more interest in (additional) care services than less anxious and depressed persons (4, 6). This could result from the fact that anxious and depressed people have a greater interest in managing their psychological burden associated with PS and also experience a lower threshold for accessing services because they might have already received psychological help.

The finding that people with higher skin-related illness identity were more interested could maybe be explained by a greater interest in managing their illness in this group.

In this context, however, it is interesting to note that less stressed PS-patients were more interested in participation in a short psychological intervention than more stressed PS-patients. This is in contradiction with studies showing that emotionally burdened individuals usually use more clinical help (31, 32). However, in our study, these results might be explained by the fact that people who are busy participating in other programs during their stay at the rehabilitation clinic do not want to additionally take part in a psychological intervention that after all included 8 sessions of about 45 min plus homework (22).

Patients, who reported to be less mindful, were also more interested in participation than patients with higher scores regarding mindfulness. This result can possibly be explained by the fact that patients scoring low on mindfulness hoped for an increase of their mindfulness by participation in the intervention and regarded this as necessary to manage their disease.

Interestingly, also the belief that nutrition contributed to the disease differed between patients with and without interest in a short psychological intervention: Patients, who were interested in participation in the intervention, more often believed that their nutrition impacted their skin disease. With this regard, it is possible that patients who believe that they have aggravated their skin disease by an unhealthy lifestyle would now like to change this by participating in any additional program offered at the clinic (e.g., nutrition counseling/stress management/sport activities).

The result that rather younger patients were interested in a short psychological intervention fits to the result of a former study including patients with breast cancer. Here, also younger persons were more likely to participate in a psychological intervention than older persons (33). However, it has to be stated that interest in and actual participation are two different things (also see below).

Contrary to our expectation, patients with and without interest in a short psychological intervention did not differ regarding the severity of PS. This finding is in line with the results of a former study, in which interest in participation in a patient education program for parents of children with atopic dermatitis was also not related to the skin status of the children (34). When interpreting this result, it has to be kept in mind that psychological burden is not linear to the severity of the skin condition in PS-patients (35). This could also be shown in this study as the severity of the PS neither significantly correlated with anxiety nor with depression (*p* > 0.05).

Dermatologists working at a rehabilitation clinic can profit from knowing which patient characteristics contribute to interest in (further) psychological treatment during the stay at the clinic as this can lead to especially addressing patients without interest in order to raise their motivation. According to the transtheoretical model of health behavior (36) patients in different stages of behavior change profit from different information. It can thus, e.g., be helpful to provide empirical data on the relationship between psychological factors and PS as well as on the effects of psychological interventions in dermatological patients to a certain group of PS-patients in order to improve the decision making of patients who are not interested in psychological interventions in the first place.

However, before clear recommendations can be given from the results, they should be replicated in a larger sample, also comprising outpatients. Moreover, future prospective studies should investigate whether there are certain characteristics, which differentiate between patients who actually take part in a psychological intervention and those who only pretend to be interested, but at the end drop out during the course of the intervention or do not participate at all.

## Data availability statement

Data will be made available by the corresponding author upon reasonable request.

## Ethics statement

The studies involving human participants were reviewed and approved by Ethics Committee at the Faculty of Medicine at the Justus-Liebig-University Gießen, Germany. The patients provided their written informed consent to participate in this study.

## Author contributions

LS: conceptualization (support), investigation (equal), methodology (support), project administration (support), formal analysis (support), visualization (lead) and writing—original (equal). ME: conceptualization (support), data curation (equal), investigation (equal), methodology (equal), project administration (support), and writing—review and editing (equal). CZ: conceptualization (support), investigation (support), project administration (support), resources (support), and writing—review and editing (equal). JK: conceptualization (lead), methodology (equal), project administration (support), resources (equal), supervision (support), and writing—review and editing (equal). CS: conceptualization (lead), data curation (equal), formal analysis (lead), methodology (equal), project administration (lead), resources (equal), supervision (lead), visualization (support), and writing—original draft (equal). All authors contributed to the article and approved the submitted version.

## Conflict of interest

CS has received speakers honoraria by Novartis in 2020 and is a consultant for Mahana Therapeutics, United States.

The remaining authors declare that the research was conducted in the absence of any commercial or financial relationships that could be construed as a potential conflict of interest.

## Publisher’s note

All claims expressed in this article are solely those of the authors and do not necessarily represent those of their affiliated organizations, or those of the publisher, the editors and the reviewers. Any product that may be evaluated in this article, or claim that may be made by its manufacturer, is not guaranteed or endorsed by the publisher.
